# Study on Chemical Diversity, Antioxidant and Antibacterial Activities, and HaCaT Cytotoxicity of *Camphora tenuipilis* (a Traditional Aromatic Plant from Xishuangbanna)

**DOI:** 10.3390/plants14223409

**Published:** 2025-11-07

**Authors:** Long Chen, Xuan Fan, Hao Qi, Shi-Guo Chen, Ren Li, Yu-Jing Liu

**Affiliations:** 1 College of Life Sciences, Nanjing Agricultural University, Nanjing 210095, Chinachenshg@njau.edu.cn (S.-G.C.); 2 Southeast Asia Biodiversity Research Institute, Chinese Academy of Sciences, Menglun 666303, China; 3 Xishuangbanna Tropical Botanical Garden, Chinese Academy of Sciences, Menglun 666303, China; 4 Yunnan International Joint Laboratory of Southeast Asia Biodiversity Conservation, Menglun 666303, China

**Keywords:** essential oil, *Camphora tenuipilis*, chemical composition, total polyphenol, antioxidant activity, antibacterial activity, cytotoxic

## Abstract

*Camphora tenuipilis*, a unique aromatic plant in the traditional Xishuangbanna dish “Duo Sheng” (raw minced meat dish), lacks scientific evidence to support its traditional use and potential application as a natural preservative/antioxidant. This study aims to fill this gap by analyzing the chemical composition and bioactivities of its leaf essential oils (EOs), verifying its traditional use, and exploring the bioactivities specific to different chemotypes. Leaf samples were collected from the Xishuangbanna Tropical Botanical Garden (XTBG), Chinese Academy of Sciences, and local markets. Gas chromatography–mass spectrometry (GC-MS) analysis identified 53 compounds, leading to the classification of the EOs into five chemotypes: linalool, geraniol, citral, elemicin, and methyl cinnamate. Notably, the elemicin-type EO (YC02, with an elemicin content of 94.56 ± 0.98%) exhibited the strongest antioxidant properties. The EOs demonstrated antibacterial activity against four foodborne pathogens: *Bacillus cereus*, *Bacillus subtilis*, *Escherichia coli*, and *Staphylococcus aureus*; except for YC04, the other EOs effectively inhibited pathogen growth to varying extents. Cytotoxicity tests revealed half-maximal inhibitory concentrations (IC_50_) for HaCaT cells ranging from 0.163 to 0.847 mg/mL. This study scientifically validates the traditional use of *C. tenuipilis* in “Duo Sheng” and supports its potential as a natural food preservative, antioxidants, and antimicrobial agents.

## 1. Introduction

*Camphora* plants are widely distributed and cultivated globally due to their rapid growth, distinctive fragrance, and substantial size [1,2]. Formerly classified under the genus *Cinnamomum*, these plants have recently been recognized as a distinct genus within the Lauraceae family, which is renowned for its medicinal value and aromatic properties [3]. Ecologically, they play a crucial role in maintaining the balance of tropical and temperate ecosystems; in terms of resource utilization, they serve as a core source of timber, medicinal materials, spices, and essential oils [4]. EOs from *Camphora* leaves are rich in monoterpenes, sesquiterpenes, esters, aldehydes, and ketones [5], components that act as plant defense against pests/diseases and exhibit antioxidant, antibacterial, anti-inflammatory, and insect-repellent effects [1,6,7], supporting their wide application in food processing, pharmaceutical and cosmetic industries [8,9].

Currently, foodborne diseases caused by microorganisms remain a major global public health challenge [10,11]. While antibiotics mitigate related risks, their misuse increases antibiotic-resistant pathogens [10,12,13]; synthetic antioxidants (e.g., BHA, BHT) in food/cosmetic industries extend shelf life [14,15] but raise toxicity concerns with prolonged use [16]. In this context, plant EOs, characterized by environmental friendliness, biodegradability, low vertebrate toxicity, and easy extraction [15,17,18], have emerged as ideal resources for safe antioxidants and antimicrobials [19,20], making the screening of plant-derived natural bioactive compounds a focal area of contemporary research [21,22].

*Camphora tenuipilis* (Kosterm.) Y. Yang, Bing Liu & Zhi Yang, a *Camphora* species distributed in valleys/gorges of southern and western Yunnan (580–2100 m altitude) [23,24,25,26] and exhibits fast growth and strong regenerative ability with prominent leaf EO content (0.39–1.02% dry weight). Owing to its distinctive leaf fragrance, it holds cultural significance in Xishuangbanna ethnic dietary traditions: the Dai, Hani, and Lahu historically use its leaves as a seasoning for meats, forming the regionally recognized intangible cultural heritage “duo sheng” (raw minced/seasoned meat). “Duo sheng” involves adding *C. tenuipilis* leaves directly to raw meat, but this raw, unsterilized dish is prone to oxidation, spoilage, and microbial infection risks—and crucially, no robust scientific evidence directly validates the safety or efficacy of *C. tenuipilis* as a “duo sheng” seasoning, which creates a critical research gap.

Existing studies confirmed *C. tenuipilis* EOs contain bioactive compounds (linalool, citral, methyl cinnamate and geraniol) [23,25,26,27], which exhibit antimicrobial, antioxidant, and anti-inflammatory activities in other plants [28,29,30]. More notably, *C. tenuipilis* is highly chemically polymorphic, with leaf EOs classified into geraniol-, linalool-, citral-, elemicin-, and farnesol-types (the most chemotypes among *Camphora* species) [23,24,25,26,27]. However, the link between its chemotype diversity and bioactivity differences remains understudied, limiting precise application of its EOs.

Based on the above, this study focuses on two core objectives to address the identified gaps: (1) systematically evaluate the antioxidant potential (via ABTS, DPPH, FRAP), antibacterial activity (against *Escherichia coli*, *Staphylococcus aureus*, *Bacillus cereus*, *Bacillus subtilis*)*,* and HaCaT cell cytotoxicity of EOs from different *C. tenuipilis* chemotypes, aiming to provide direct scientific evidence for its traditional “Duo Sheng” use and theoretical support for developing natural antioxidants and antimicrobials; (2) explore the reciprocal relationship between ethnic dietary cultural diversity (e.g., “Duo Sheng” tradition knowledge) and *C. tenuipilis* chemical diversity (e.g., EO chemotypes), offering a reference for interdisciplinary research in ethnobotany and biodiversity conservation.

## 2. Results

### 2.1. Chemical Composition Analysis of Five C. tenuipilis Essential Oils

**Figure 1:**
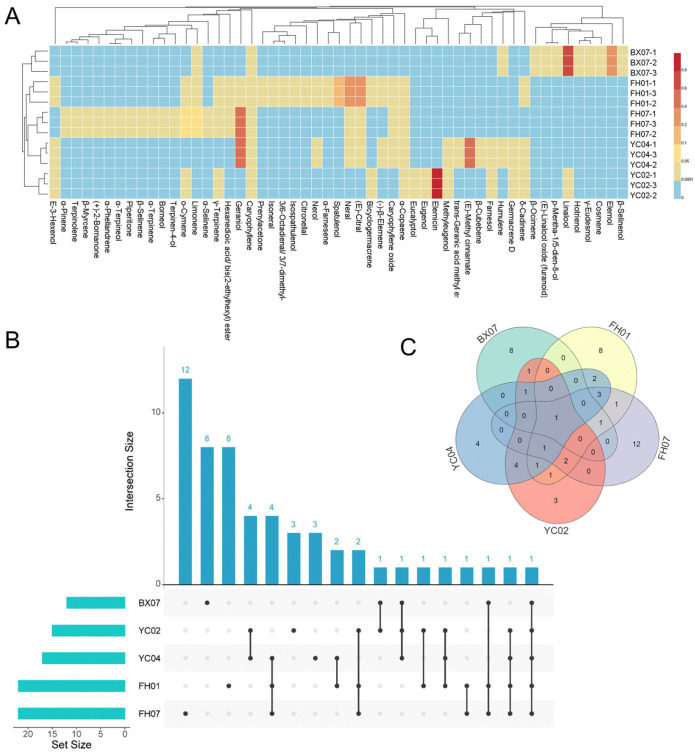
Chemical composition analysis of five *C. tenuipilis* essential oils. Ingredient heatmap (**A**) was used to display the differences in five essential oils visually. UpSet plot (**B**) illustrating shared and unique components among the five *C. tenuipilis* essential oils, the numbers in the figure represent the quantity of unique components and shared components of the EO. Venn diagram (**C**) illustrating shared and unique components among the five *C. tenuipilis* essential oils, the numbers in the figure indicate the number of shared components between the EOs.

Gas chromatography–mass spectrometry (GC-MS) analysis was performed on fifteen EO samples derived from fifteen distinct *C. tenuipilis* individuals, leading to the identification of 53 compounds, which comprise 94.73–99.27% of the total essential oil composition. It can be observed that the major components significantly among the five chemotypes (*p* < 0.05) (Table 1, Figure S1). As shown in Table 1, components of the essential oil that account for more than 5% of the oil are defined as the major constituents. It can be observed that the major chemical components differ significantly among different individuals, and these differences are more clearly illustrated in the heatmap (Figure 1A). Among them, the major chemical components of the BX07 are linalool (68.84%) and elemol (20.72%); the main components of the FH01 are (E)-citral (35.80%), neral (30.75%), and spatulenol (10.62%); the major components of the FH07 are geraniol (54.46%), limonene (7.18%), and o-cymene (6.24%); the major component of the YC02 is elemicin (94.56%); and the main components of the YC04 are (E)-methyl cinnamate (46.20%) and geraniol (46.15%).

UpSet analysis and Venn diagrams were utilized to illustrate the shared and unique components among the fifteen EO samples from *C. tenuipilis* leaves (Figure 1B,C). The results show that the components of the five essential oils exhibit significant differences. The only common component among the five oils is caryophyllene. Apart from the BX07 chemotype, α-copaene is the only component shared by the other four essential oils. Geraniol is the main shared component in the FH07 and YC04 oils, accounting for 68.84% and 46.15% of the total oil content, respectively. Based on the highest content and specific components, the *C. tenuipilis* leaf essential oils can be categorized as five distinct chemotypes: linalool-type, citral-type, geraniol-type, elemicin-type, and methyl cinnamate-type. Furthermore, only BX07 contained elemol, only YC02 contained high proportions of elemicin, only YC04 contained high proportions of (E)-methyl cinnamate, only FH01 contained high proportions of spatulenol, and only FH07 contained α-pinene. These minor components can serve as auxiliary identification characteristics to further confirm chemotype classification.

### 2.2. Total Polyphenol Content and Antioxidant Capacity

The essential oil from the leaves of five chemotypes of *C. tenuipilis* chemotypes exhibited significant variations in total polyphenol content. Table 2 shows that YC02 exhibited the highest total phenolic content, at 239.4 ± 6.87 mg GAE/g DW, followed by FH01 with 153.8 ± 1.58 mg GAE/g DW, and FH07 with 113.4 ± 1.87 mg GAE/g DW. The antioxidant capacity of the essential oils was evaluated using three methods: ABTS radical cation scavenging activity, DPPH radical scavenging activity, and FRAP. The antioxidant capacities of essential oils, measured by ABTS, DPPH, and FRAP assays, were reported in Trolox equivalents (TE). Table 2 presents the ABTS radical cation scavenging activity values as follows: BX07 (20.82 ± 1.75 µmol TE/g DW), FH01 (52.09 ± 0.66 µmol TE/g DW), FH07 (62.33 ± 0.49 µmol TE/g DW), YC02 (57.27 ± 0.82 µmol TE/g DW), and YC04 (45.33 ± 0.60 µmol TE/g DW). FH07 exhibited the highest antioxidant activity as measured by the ABTS assay. The DPPH radical scavenging activity yielded values of 25.65 ± 0.57, 75.99 ± 1.65, 49.55 ± 1.01, 83.75 ± 0.15 and 40.44 ± 0.85 µmol TE/g DW in BX07, FH01, FH07, YC02, and YC04, respectively. YC02 exhibited the highest antioxidant activity in the DPPH assay, differing from the ABTS test results. However, the best antioxidant activity detected via FRAP is also from YC02. The essential oils from the five distinct chemotypes of *C. tenuipilis* leaves exhibit significant variations in chemical composition and content. To comprehensively assess the antioxidant capacity of the essential oils, the total antioxidant capacity (TAC) was used to integrate and evaluate their antioxidant properties. The results showed that in the in vitro experimental system of this study, YC02 (with an elemicin content as high as 94.56%) exhibited significant antioxidant activity (TAC). The ranking of TAC of the five essential oils was as follows: YC02 (97.29%) > FH01 (83.83%) > FH07 (72.73%) > YC04 (61.86%) > BX07 (28.90%). Notably, this ranking was consistent with that of the essential oils’ total phenolic content.

**Figure 2:**
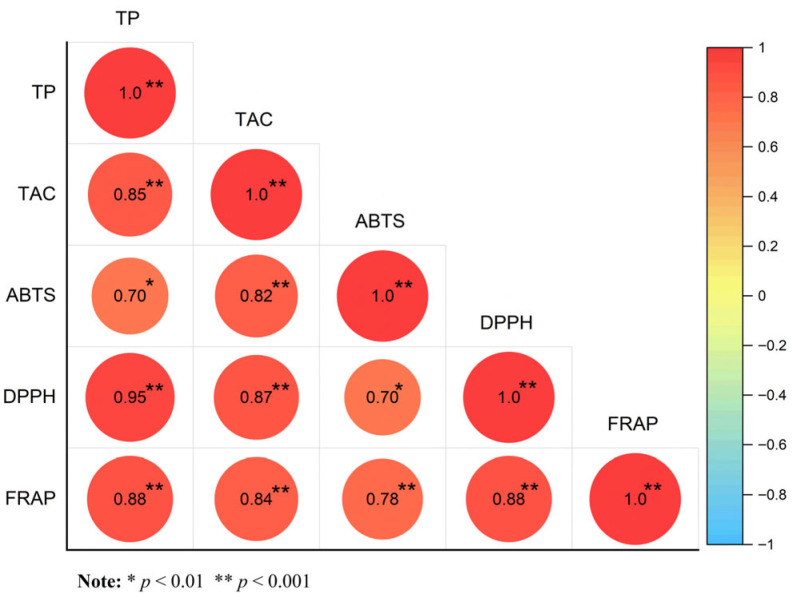
A Pearson correlation plot was generated to evaluate the relationships between total polyphenol (TP) content, total antioxidant capacity (TAC), FRAP (ferric reducing antioxidant power), DPPH (2,2-diphenyl-1-picrylhydrazyl), and ABTS (2,2′-azino-bis (3-ethylbenzothiazoline-6-sulfonic acid)). The Pearson correlation coefficient was calculated to determine the strength and direction of the linear relationships among these variables. An asterisk (*) denotes significant differences, with * indicating *p* < 0.05 and ** indicating *p* < 0.01.

A Pearson correlation analysis was performed to assess the relationship between total phenolic content and antioxidant capacity (Figure 2). The study found a strong positive correlation between the total phenolic content and antioxidant capacity of *C. tenuipilis* leaf essential oils, with a correlation coefficient of 0.95 for DPPH TE equivalents and 0.85 for the total antioxidant capacity index (TAC) relative to total phenolic content. This indicates that phenolic compounds predominantly contribute to the essential oil of the examined *C. tenuipilis*.

### 2.3. Antibacterial Activity

**Figure 3:**
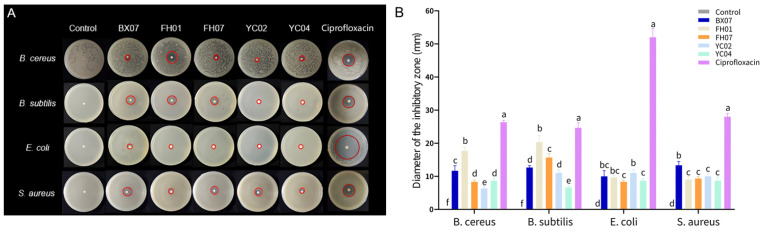
Antibacterial activity of five *C. tenuipilis* essential oils. DIZ images (**A**) and statistical data (**B**) for *C. tenuipilis* essential oils tested against *B. cereus*, *B. subtilis*, *E. coli*, and *S. aureus*. The red circle in the figure A represents the size of the inhibition zone. Discs had a diameter of 6 mm, with values expressed as means ± standard deviations (*p* < 0.05). Means in a group with different letters indicate statistical significance (*p* < 0.05).

The antibacterial effects of various *C. tenuipilis* leaf essential oils on *B. cereus*, *B. subtilis*, *E. coli*, and *S. aureus* were assessed using DIZ, MIC, and MBC methods (Figure 3; Table 3). The study found varying susceptibility levels of *B. cereus*, *B. subtilis*, *E. coli*, and *S. aureus* to the five essential oils, with inhibition halos measuring between 6.33 and 20.33 mm. The 5 μg ciprofloxacin inhibition zones against four bacteria range from 24.67 to 52 mm, with *E. coli* showing the highest sensitivity. Conversely, bacterial growth covered the filter paper in the control group. Gram-negative bacteria (*E. coli*) exhibited lower susceptibility to *C. tenuipilis* leaf essential oils compared to Gram-positive bacteria (*B. cereus*, *B. subtilis*, and *S. aureus*). The minimum inhibitory concentration (MIC) of ciprofloxacin against the four bacteria is all lower than 0.5 µg/mL. In contrast, the MIC of the essential oils against the four bacteria mostly ranges from 3.13 to 25 µL/mL.

Preliminary screening via Diameter of Inhibition Zone (DIZ) determination revealed that, except YC04, the other four chemotype essential oils exhibited inhibitory effects against *Bacillus cereus*, *B. subtilis*, *Escherichia coli*, and *Staphylococcus aureus*. Among these, BX07 (rich in linalool) and FH01 (rich in citral) exhibited a broader antibacterial spectrum, showing strong inhibitory activity against common bacteria with particularly prominent inhibitory activity against Gram-positive bacteria, which indicates their potential as natural antimicrobial agents. Furthermore, FH01 showed the strongest antimicrobial effects against *B. cereus* and *B. subtilis*, with inhibition zones diameters all exceeding 17 mm and low MIC and MBC values. BX07 displayed moderate antibacterial capacity against all four bacteria, with inhibition zone diameters ranging from 10 to 13.33 mm; FH07 and YC02 had limited antibacterial effectiveness; whereas YC04, despite containing a relatively high level of (E)-methyl cinnamate and geraniol, showed extremely weak antibacterial activity, with its MBC values exceeding 12.5 μL/mL.

### 2.4. Cytotoxicity to Human Keratinocyte (HaCaT)

**Figure 4:**
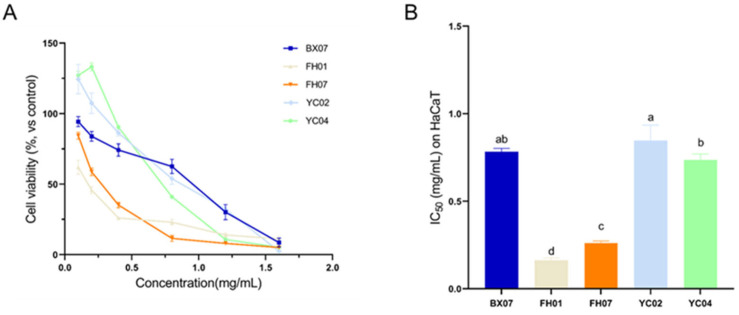
Cytotoxicity of *C. tenuipilis* EOs on human keratinocytes (HaCaT). Panel (**A**) shows the cytotoxic effects of *C. tenuipilis* EOs on HaCaT cells, while panel (**B**) presents the IC_50_ values, which represent the concentration required to inhibit 50% of cell viability. All data are presented as means ± standard deviations (*p* < 0.05). Statistically significant differences are indicated by different letters within the same column, where values with different letters differ significantly (*p* < 0.05), highlighting the varying degrees of cytotoxicity among the different EOs tested.

In this study, the CCK-8 assay was employed to evaluate the cytotoxicity of leaf essential oils (EOs) of *C. tenuipilis* to human keratinocytes (HaCaT cells). The cytotoxicity of the five *C. tenuipilis* leaf EOs against HaCaT cells was determined (Figure 4A), and curve fitting was performed (Figure S2). For the control groups: the cell viability of the negative control group (with 0.1% dimethyl sulfoxide (DMSO) as the solvent) was 99.1 ± 2.35%, while that of the positive control group (with 10% DMSO) was 13.82 ± 2.21%.

The results showed that the half-maximal inhibitory concentration (IC_50_) values of the five EOs against HaCaT cells ranged from 0.163 to 0.847 mg/mL (Figure 4B), which classifies these EOs as moderately toxic. Among them, FH01 (IC_50_ = 0.163 ± 0.013 mg/mL) and FH07 (IC_50_ = 0.261 ± 0.012 mg/mL) exhibited relatively higher cytotoxicity, and their cytotoxicity was within the safety threshold at a concentration of 0.1 mg/mL. In contrast, the other three EOs (BX07, YC02, YC04) showed significantly lower cytotoxicity, with IC_50_ values of 0.782 ± 0.02 mg/mL, 0.847 ± 0.088 mg/mL, and 0.736 ± 0.034 mg/mL, respectively. Notably, YC02 and YC04 also exhibited a slight cell proliferation-promoting effect at low concentrations (e.g., 0.1–0.4 mg/mL), with cell viability exceeding 84% and even reaching over 125%. Their biocompatibility was the best among the five chemotypes.

## 3. Discussion

For the first time, this study identified five chemotypes of *C. tenuipilis*, a plant used in “Duosheng” (a traditional raw meat dish) in the Xishuangbanna region, and conducted a systematic analysis of the chemical composition, antioxidant activity, antibacterial activity, and cytotoxicity of the leaf essential oils (EOs) from these five chemotypes. The results not only provide scientific evidence for the traditional use of *C. tenuipilis* in “Duosheng” but also lay a foundation for the development of natural antioxidants and antibacterial agents.

Regarding chemical composition, a total of 53 compounds were identified in the EOs of the five chemotypes, which can be classified into five main chemotype categories: linalool-type, citral-type, geraniol-type, elemicin-type, and methyl cinnamate-type. This significant chemical diversity highlights the plant’s ecological adaptability and its potential to produce various bioactive substances. The rich monoterpenes and sesquiterpenes (e.g., linalool, elemol) not only highlight the plant’s value as a reservoir of natural bioactive compounds but also provide a chemical basis for its traditional use in “Duosheng”. “Duosheng” is a raw meat dish susceptible to microbial spoilage and oxidation [31], and the antibacterial properties of linalool, the flavor-enhancing effect of citral [32,33,34], and the synergistic antioxidant effect of sesquiterpenes [35] precisely meet the demands for food preservation, freshness retention, and flavor enhancement in traditional diets. This finding is the first to link the “Duosheng” dietary custom with plant components from a chemical perspective, scientifically validating this traditional practice. Notably, previous studies have demonstrated the antioxidant activity of elemicin in *Myristica fragrans*, where elemicin also serves as the main active ingredient [36]. In contrast, the elemicin content in the leaf EOs of the YC02 chemotype reaches as high as 94.56%, a high content rarely observed in *Cinnamomum* plants, and this is the key reason for YC02’s excellent antioxidant capacity [37,38]. Among the leaf EOs of the five *C. tenuipilis* chemotypes, only caryophyllene is a common compound. This suggests that a divergent biosynthetic pathway driven by ecological or genetic factors may exist in *C. tenuipilis*, providing a valuable system for future research on biosynthesis and metabolic regulation.

In terms of antioxidant activity, YC02 exhibited the highest total phenol content (239.4 mg GAE/g dry weight) and total antioxidant capacity (TAC = 97.29%) among the five chemotypes. Additionally, there was a strong positive correlation between polyphenol content and antioxidant activity (r = 0.85), which is consistent with findings in *Perilla frutescens* [39]. This further supports the core role of phenolic compounds in free radical scavenging and lipid oxidation inhibition and indicates the potential application value of YC02 in food preservation and anti-aging cosmetics [37,40,41,42]. In this study, differences were observed in the antioxidant activity rankings obtained using three detection methods (ABTS, DPPH, and FRAP). This may be related to the different mechanisms involved in each method, namely electron transfer, hydrogen atom donation, and metal reduction, respectively [43]. Future studies can further clarify this mechanism through molecular docking, oxidative stress-related gene expression analysis, and other approaches [44]. Furthermore, in addition to phenolics, flavonoids and terpenoids also contribute to the total antioxidant activity [45], indicating that the antioxidant effect of EOs stems from the synergistic action of multiple components rather than the independent effect of a single compound.

Significant differences exist in the content and type of core components in the leaf EOs of the five *C. tenuipilis* chemotypes (YC02, FH01, BX07, FH07, YC04), which directly lead to variations in their antibacterial activity against four target bacteria: *B. cereus*, *B. subtilis*, *E. coli*, and *S. aureus*. Moreover, interactions (e.g., antagonism) between components in some chemotypes may affect the overall antibacterial efficacy. Specifically, the elemicin content in the leaf EOs of the YC02 chemotype exceeds 94%, with no interference from other high-abundance components, and it exhibits outstanding antibacterial activity against *B. subtilis*, *B. cereus*, and *E. coli*. Although the chemical structure of elemicin is somewhat complex, and systematic research on its bioactivity and potential applications remains limited, it is hypothesized that its core mechanism of action may revolve around bacterial cell walls/membranes and metabolic processes; the specific mechanism requires further clarification in subsequent studies. The FH01 chemotype contains more than 60% citral, a well-recognized broad-spectrum antibacterial component that exhibits antibacterial activity against various bacteria and fungi [29,46], with a particularly significant inhibitory effect on Gram-positive bacteria (e.g., *Bacillus* spp.). Its mechanism of action involves disrupting bacterial cell membrane integrity and inhibiting enzyme activity. The BX07 chemotype shows moderate-to-strong broad-spectrum antibacterial activity against all four bacteria. Its main component, linalool, accounts for 68.64% of the EO and is the primary contributor to antibacterial activity. Linalool exhibits dual-spectrum antibacterial activity against both Gram-positive and Gram-negative bacteria, acting by altering bacterial cell membrane permeability [47,48,49]. Additionally, elemol (19.99~21.45%) in BX07 exerts an auxiliary effect of enhancing antibacterial activity, ultimately endowing this chemotype with broad-spectrum moderate antibacterial activity. The FH07 chemotype contains more than 53% geraniol, and high concentrations of geraniol exhibit significant antibacterial activity against Gram-positive bacteria (e.g., *B.* *subtilis*). This may be related to differences in bacterial cell wall structure: Gram-positive bacteria have a cell wall composed solely of a thick peptidoglycan layer (without an outer membrane), resulting in significantly higher permeability to hydrophobic compounds (e.g., geraniol) compared to Gram-negative bacteria (which have a thin peptidoglycan layer + outer membrane) [50,51]. Thus, geraniol can more easily reach and act on Gram-positive bacteria. Although the YC04 chemotype also contains a high content of geraniol (approximately 46%) and additionally a high content of methyl cinnamate (approximately 46%), its antibacterial activity is significantly lower than that of FH07 (dominated by geraniol alone). This suggests a potential antagonistic interaction between geraniol and methyl cinnamate, indicating that the antibacterial efficacy of EOs is not determined by a single component but by complex synergistic or antagonistic interactions among internal components [52]. The complexity of such component interactions highlights the importance of prioritizing the overall component composition of EOs when developing natural antibacterial agents. Future studies can further analyze key active components and their interaction mechanisms using techniques such as fractional distillation-GC/MS and microencapsulation [53,54].

To date, numerous studies have confirmed that EOs from Lauraceae plants possess antibacterial activity, exhibiting inhibitory effects against common bacteria (e.g., *Escherichia* *coli*, *Staphylococcus aureus*, *Bacillus subtilis*, *Pseudomonas aeruginosa*) and fungi (e.g., yeasts, *Botrytis cinerea*, *Candida albicans*) [50,55,56,57,58,59]. Specifically, the minimum inhibitory concentration (MIC) of *Litsea cubeba* EO against *B. subtilis* is 50 mg/mL; in contrast, the MIC range of C. tenuipilis EO against the two *Bacillus* species is 3.13–12.5 μL/mL, indicating significantly superior antibacterial activity compared to *L. cubeba* EO [60]. Another study showed that the MIC of *Cinnamomum camphora* EO against both *E. coli* and *S. aureus* is 6.25 mg/mL, with a minimum bactericidal concentration (MBC) of 25 mg/mL for both [61], an antibacterial effect comparable to that of *C. tenuipilis* EO observed in this study. In summary, consistent with most Lauraceae plant EOs, *C. tenuipilis* EO also exhibits excellent antibacterial activity.

Keratinocytes account for approximately 95% of epidermal cells and are the main cell type in human epidermal tissue. The *C. tenuipilis* EOs investigated in this study are derived from a traditionally edible plant, and their initial contact sites with the human body are the oral and gastrointestinal mucosal epithelia. Mucous membranes and skin both belong to stratified epithelial tissues and share high structural similarity. Based on this, human keratinocytes (HaCaT cells) serve as a standardized epithelial cell model, and their toxicity data can provide key references for the preliminary safety risk assessment of EOs [62]. Therefore, HaCaT cells were selected for cytotoxicity experiments in this study. Toxicity assessment results showed that the half-maximal inhibitory concentration (IC_50_) of leaf EOs from the five *C. tenuipilis* chemotypes against HaCaT cells ranged from 0.163 to 0.847 mg/mL, generally falling into the moderate toxicity category. However, significant differences in toxicity strength were observed among different chemotypes, providing core evidence for the precise adaptation of EOs to subsequent application scenarios and safety risk management. Notably, in vitro cytotoxicity results should be distinguished from actual edible safety: *C. tenuipilis*, a spice plant used in “Duosheng” (a traditional raw minced meat dish) by ethnic groups such as the Dai and Hani in Xishuangbanna, has an actual intake concentration in the diet that is far lower than its in vitro toxic IC_50_ range (0.163–0.847 mg/mL), and this intake concentration is consistent with the 0.1 mg/mL safety threshold (below the toxic threshold) set in our experiment. Furthermore, after the essential oil of this plant enters the human body, it may undergo metabolism in the digestive system, leading to reduced activity; the human body’s own detoxification mechanism can further diminish its toxicity. Meanwhile, the long history of its consumption in ethnic traditions has empirically confirmed its safety under regular dietary exposure levels. The current in vitro toxicity data only serves as a preliminary risk indicator and cannot be directly equated to in vivo toxic effects [63]: after entering the human body, EOs may undergo metabolic processes in the digestive system to reduce their activity, and the body’s own detoxification mechanisms can further weaken their toxicity. Therefore, accurate edible safety evaluation requires subsequent acute/subchronic toxicological experiments using animal models to verify differences in toxic responses at different concentrations, clarify the quantitative relationship between toxicity thresholds and actual intake, and combine metabolomic analysis to reveal in vivo detoxification pathways. This will provide more scientific support for the edible safety and risk management of EOs.

Based on the above findings, an application selection system for *C. tenuipilis* EOs can be established through correlation analysis between chemical components and bioactivities: YC02 is preferentially suitable for scenarios requiring antioxidant properties, while FH01 and BX07 are more appropriate for antibacterial-oriented applications. The high chemical diversity and multifunctional characteristics of *C. tenuipilis* provide valuable materials for its variety breeding, metabolic regulation, and sustainable resource utilization. Future studies will combine multi-omics analysis (transcriptomics and metabolomics) and skin/gut microbiota models to further explore its mechanism of action [50,51], promoting the transformation of this traditional plant resource from a carrier of ethnic dietary culture to a high-value natural product. This holds dual significance in protecting ethnic cultural heritage and exploring scientific and economic value.

Additionally, this study provides a typical case for understanding the coevolutionary relationship between biodiversity and cultural diversity [64]. The chemical diversity of *C. tenuipilis* at the chemotype level provides a material basis for its adaptation to different ecological environments and human utilization patterns [65]. In the long-term practices of ethnic minorities such as the Dai, Hani, and Lahu in Xishuangbanna, the preparation process of “Duosheng” has implicitly promoted the identification, screening, and protection of the chemical diversity of *C. tenuipilis*. Traditional flavor preferences and cooking practices essentially constitute a culture-driven germplasm resource protection mechanism, which not only preserves local dietary culture but also promotes the in-situ conservation of the plant’s chemical diversity [66,67]. Therefore, the multiple chemotypes of *C. tenuipilis* can be regarded as vivid evidence of the symbiotic interaction between biodiversity (genetic and chemical characteristics) and cultural diversity (ethnic dietary traditions). Protecting such traditional knowledge is of great significance for maintaining species genetic diversity and ecological functions [68].

## 4. Materials and Methods

### 4.1. Plant Materials and Bacterial Strains

Samples of *C. tenuipilis* were collected from the Xishuangbanna Tropical Botanical Garden (XTBG), Chinese Academy of Sciences (located in Menglun Township, Yunnan province, China), and local markets in Yunnan Province. These samples were collected over a four-week period from July to August 2022 and subsequently stored in the shaded at room temperatures (20–25 °C) for two months. All plant materials were identified by Mr. Zhou Shishun from XTBG, and voucher specimens for the fifteen individuals (corresponding to the five chemotypes: BX07, FH01, FH07, YC02, and YC04) were deposited in the XTBG herbarium (HITBC).

*B. cereus* ATCC 14579, *B. subtilis* ATCC 6633, *E. coli* ATCC 25922 and *S. aureus* ATCC 25923 were all obtained from the Culture Collection Center of the College of Life Sciences, Nanjing Agricultural University. All strains were stored at −80 °C in Luria–Bertani (LB) broth with 25% glycerol (*v*/*v*). Prior to each experiment, the test strains were incubated in LB broth at 37 °C with shaking for 18 h.

### 4.2. Isolation of Essential Oils

Dried leaf samples were collected from fifteen distinct *C. tenuipilis* individuals (distributed across multiple locations in Xishuangbanna, including Xishuangbanna Tropical Botanical Garden and local markets) and ground into powder separately. For each individual plant, a 100 g aliquot of the powdered sample was mixed with 1000 mL of distilled water, and essential oils were extracted via steam distillation using a Clevenger instrument. After boiling and extraction for 3 h, the extracted liquid was dried with anhydrous sodium sulfate to obtain *C. tenuipilis* leaf essential oils, which were then stored in a brown essential oil bottle at 4 °C refrigerator for subsequent analysis. Each sample was numbered as BX07-1, BX07-2, BX07-3, FH01-1, FH01-2, FH01-3, FH07-1, FH07-2, FH07-3, YC02-1, YC02-2, YC02-3, YC04-1, YC04-2 and YC04-3, for component content analysis.

### 4.3. Analysis of the Essential Oil Components

Essential oil samples were diluted with n-hexane. Gas chromatography–mass spectrometry (GC-MS) was conducted using an Agilent Technologies 7890A-7000B GC-MS system, featuring an HP-5MS capillary column (50 mm × 0.32 mm × 0.52 μm). The injector and detector (MS transfer line) temperatures were set at 250 °C. The column operates with helium as the carrier gas at a flow rate of 1 mL/min and a transfer line temperature of 250 °C. The acquisition mass range was set from 45 to 500 *m*/*z* with an ionization voltage of 70 eV. The column temperature is initially maintained at 40 °C for 2 min, then increased linearly to 160 °C at 3 °C/min, followed by a rise to 250 °C at 20 °C/min, and held for 10 min. A manual injection of 0.2 µL of the diluted essential oil sample is performed. Components are identified by comparing calculated experimental GC retention indices for n-alkanes C_7_-C_30_ under identical conditions with those in the NIST Standard Reference Database [69]. The relative percentages of the various components of the essential oil are determined using the normalization method to calculate the peak area. For each essential oil sample, three replicates (labeled as −1, −2, −3) were conducted to verify the reproducibility and stability of the chemical composition analysis results.

### 4.4. Determination of Total Polyphenolic Content

The Folin–Ciocalteu method was used to measure the total polyphenol (TP) content in the essential oils of *C. tenuipilis* leaves [39,70]. In summary, 120 µL of 20% (*v*/*v*) Folin–Ciocalteu reagent was introduced to each well of a 96-well plate, followed by 20 µL of various essential oil samples. The blank control wells received 20 µL of distilled water. Samples were incubated at room temperature for 5 min, followed by the addition and mixing of 20 µL of 7% Na_2_CO_3_ solution. The plate was then incubated at 40 °C for 20 min until the solution turned blue. After the reaction, the absorbance of each well was measured at 760 nm using a Tecan Infinite M200 Multi-Detection Plate Reader (Thermo Fisher Scientific, Waltham, MA, USA). The polyphenolic content of the samples was measured using a gallic acid standard curve and reported as milligrams of gallic acid equivalents per gram of dry matter (mg GAE/g DM). Each sample was measured three times, with results presented as the mean ± standard deviation (SD) of these independent measurements.

### 4.5. Determination of Total Antioxidant Capacity

The total antioxidant capacity (TAC) [71] was evaluated using three different methods: 2,2′-azino-bis-3-ethylbenzthioazoline-6-sulfonic acid (ABTS) radical cation scavenging activity, 2,2-diphenyl-1-picrylhydrazyl (DPPH) radical scavenging activity, and ferric reducing antioxidant power (FRAP) assay.

#### 4.5.1. ABTS Radical Cation Scavenging Activity

The ABTS antioxidant capacity was assessed using an ABTS assay kit (CominBio, Suzhou, China). In a 96-well plate, 10 µL of each sample or ethanol (as a blank control) was combined with 190 µL of the reaction solution. Absorbance at 734 nm was recorded within 10 min using the Tecan Infinite M200 Multi-Detection Plate Reader (Thermo Fisher Scientific, Waltham, MA, USA). ABTS antioxidant activity was measured in µmol Trolox equivalents (TE) per gram of dry weight [72], utilizing the calibration equation y = 0.7021x − 0.0012 (R^2^ = 0.9985), where x denotes Trolox concentration (µmol/mL) and y is the change in optical density (∆OD) between the sample and the blank.

#### 4.5.2. DPPH Radical Scavenging Activity

The antioxidant activity of the essential oils was determined using a DPPH assay kit (CominBio, Suzhou, China). A 20 µL aliquot of each sample or ethanol (blank control) was mixed with 380 µL of DPPH solution and incubated in the dark at room temperature for 20 min. Subsequently, 200 µL of the reaction mixture was transferred to a 96-well plate, and the absorbance at 515 nm was measured using the Tecan Infinite M200 Multi-Detection Plate Reader (Thermo Fisher Scientific, Waltham, MA, USA). The DPPH antioxidant activity was expressed as µmol Trolox equivalents (TE) per gram of dry weight [72], based on the calibration curve (y = 0.7072x − 0.0081, R^2^ = 0.9977), where x is the Trolox concentration (µmol/mL) and y represents ∆OD = OD sample–OD blank.

#### 4.5.3. Ferric Reducing Antioxidant Power

The FRAP assay (CominBio, Suzhou, China) was used to evaluate the ferric reducing antioxidant power (FRAP) of the essential oils. A 10 µL aliquot of each sample or ethanol (blank control) was mixed with 190 µL of FRAP reagent in a 96-well plate, and the absorbance at 593 nm was measured using the Tecan Infinite M200 Multi-Detection Plate Reader (Thermo Fisher Scientific, Waltham, MA, USA). The results were compared to a Trolox standard curve to determine the total antioxidant capacity of the samples [72]. The standard curve equation was y = 1.2416x + 0.0134, R^2^ = 0.9996, where x represents the Trolox concentration (µmol/mL) and y corresponds to ∆OD = OD sample–OD blank.

All measurements were performed in triplicate, with each sample being tested in three independent experiments. The total antioxidant capacity (TAC) was calculated using the formula TAC = [(sample score/best score) × 100]%, and the average value from the three independent experiments was used for analysis.

### 4.6. Antibacterial Activity Assay

The antibacterial activity of these essential oils was evaluated using the disc diffusion method with slight modifications [73]. A 2 mL bacterial suspension (approximately 10^7^–10^8^ CFU/mL) was added to cooled LB agar medium, mixed gently, and poured into the test plate. Following solidification, antimicrobial discs were placed on the test plate after disinfection. A 6 mm disc was treated with 3 μL of *C. tenuipilis* essential oil and incubated at 37 °C for 24 h. The inhibition zone diameter was measured with a caliper (Airaj, Tsingtao, China). Susceptibility disks containing 5 μg of ciprofloxacin were added as the positive control, and sterile water served as the negative control.

The microdilution method was used to determine the MIC and MBC values of five essential oils against *B. cereus*, *B. subtilis*, *E. coli*, and *S. aureus* [74]. Briefly, dimethyl sulfoxide (DMSO) and LB broth were used to gradient-dilute the essential oil concentration, the essential oils were dissolved in LB broth containing bacterial suspensions at 10^5^ CFU/mL, and serial twofold dilutions were prepared to achieve concentrations of 25, 12.5, 6.25, 3.125, 1.56, 0.78, 0.39 and 0.195 mg/mL. Different concentrations of ciprofloxacin solution were added to substitute the essential oil as the positive control, while sterile water was used to replace the bacterial suspension as the negative control. The 96-well plates were then incubated at 37 °C for 24 h. After incubation, 40 μL of 0.5 mg/mL p-iodonitrotetrazolium violet (INT) solution was added in each tube and further incubated for 30 min at 37 °C. The color change in each well was then checked. A pink color indicates bacterial growth. The essential oil concentration corresponding to no color change of the dye is considered the minimum inhibitory concentration (MIC) of the essential oil. A 40 μL sample from the mixture showing no visible bacterial growth was plated on LB agar and incubated at 37 °C for 24 h. The minimum bactericidal concentration (MBC) of the essential oil is defined as the concentration at which no bacterial growth is observed on the agar plate.

### 4.7. Cytotoxicity Analysis

HaCaT human immortalized keratinocytes were maintained in Dulbecco’s Modified Eagle Medium (DMEM) supplemented with 1% penicillin-streptomycin and 10% fetal bovine serum (FBS) at 37 °C in a humidified atmosphere with ≥95% humidity and 5% CO_2_. The cytotoxicity of the essential oils was assessed using the Cell Counting Kit-8 (CCK-8) assay (CY-TOCH, Shanghai, China) [75]. Cells (100 μL, 9000–12,000 cells/well) were seeded into 96-well plates and incubated for 24 h, followed by 24 h of incubation with five different concentrations of essential oils, which were diluted using a 0.1% DMSO solution. 10% DMSO served as positive control, while 0.1% DMSO functioned as the negative control [76,77]. Subsequently, each well received 10 μL of CCK-8 reagent. The plates were incubated at 37 °C for 2 h and the absorbance at 450 nm was measured using the Tecan Infinite M200 Multi-Detection Plate Reader (Thermo Fisher Scientific, Waltham, MA, USA) to determine the concentration of the water-soluble formazan dye reduction. Cell viability was normalized to the cells treated with the carrier under the same conditions. The test concentration range for each essential oil was from 0.1 mg/mL to 1.6 mg/mL, with a total of 6 concentrations (0.1, 0.2, 0.4, 0.8, 1.2, and 1.6 mg/mL). Each test was performed in triplicate. The cytotoxicity results were expressed as the half-maximal inhibitory concentration (IC_50_) value.

### 4.8. Statistical Analysis

All data were obtained at least in triplicate and represented by the mean ± standard deviation (SD). SPSS 27 software was utilized for multiple comparisons using one-way ANOVA and the least significant difference (LSD) test, considering *p* < 0.05 as statistically significant. Spearman’s correlation matrix analysis was performed using Origin 2023 software. The IC_50_ value was determined by non-linear regression analysis based on three replicates from cytotoxicity assays.

## 5. Conclusions

Taking *Camphora tenuipilis*, a traditional edible spice from Xishuangbanna, as the subject, this study focused on two core objectives, namely verifying its traditional edible use and exploring the relationship between chemical diversity and bioactivity, and systematically analyzed the chemical composition, bioactivity, and safety of its leaf essential oils (EOs). First, GC-MS analysis confirmed that the leaf EOs of this plant contained 5 stable chemotypes, with a total of 53 compounds identified; each chemotype possessed exclusive characteristic marker components. This chemical diversity not only confirms the genetic differentiation characteristics of EOs from *Camphora* plants but also provides a molecular basis for the accurate classification of *C. tenuipilis* chemotypes and resource identification. Second, bioactivity evaluation revealed a clear correlation between chemical composition and functional activity: YC02 and YC04exhibited the strongest antioxidant activity; FH01 showed the strongest inhibitory activity against Gram-positive bacteria (e.g., *Staphylococcus aureus*, *Bacillus cereus*), while BX07 and FH07 displayed broad-spectrum moderate antibacterial activity. These results clarified the functional priority that elemicin/(E)-methyl cinnamate dominates antioxidant activity, while spatulenol enhances antibacterial activity. Furthermore, HaCaT cell cytotoxicity tests verified its edible safety: all 5 chemotype EOs belonged to the moderate toxicity category. Among them, YC02 and YC04 exhibited a slight cell proliferation-promoting effect at low concentrations (0.1–0.4 mg/mL) and had the best biocompatibility; additionally, all chemotypes showed concentrations below the safety threshold at 0.1 mg/mL, which matched the actual edible dosage of *C. tenuipilis* leaves in local “Duosheng” (raw minced meat dish), providing scientific safety support for its traditional use. In conclusion, this study for the first time filled the gap in the chemical and functional research on *C. tenuipilis* as an edible spice, offering an academic reference for the classified utilization of *Cinnamomum* plant resources and laying a foundation for the development of natural food preservatives and antioxidants using superior chemotypes. Future studies may combine transcriptomics and metabolomics to elucidate the mechanism of chemotype differentiation, verify the targets of active components via molecular docking, clarify in vivo toxicity through animal experiments, and explore microencapsulation technology to improve EO stability, thereby promoting its industrial transformation and sustainable utilization.

## Figures and Tables

**Table 1:** Chemical composition of five *C. tenuipilis* essential oils.

No.	Compound	RT	RI	Area (%)				
				BX07	FH01	FH07	YC02	YC04
1	(E)-3-Hexenol	5.86	851	-	0.10 ± 0.01	-	0.05 ± 0.01	0.13 ± 0.01
2	*α*-Pinene	8.55	930	-	-	0.17 ± 0.01	-	-
3	Prenylacetone	10.85	985	-	1.10 ± 0.06	-	-	-
4	*β*-Myrcene	11.02	990	-	-	1.17 ± 0.21 ^g^	-	-
5	*α*-Phellandrene	11.49	1001	-	-	4.86 ± 0.43	-	-
6	*α*-Terpinene	12.04	1014	-	-	0.92 ± 0.05	-	-
7	o-Cymene	12.40	1024	-	0.16 ± 0.01	6.24 ± 0.11 ^c^	0.04 ± 0.01	-
8	Limonene	12.57	1026	0.08 ± 0.01	0.18 ± 0.02	7.18 ± 0.09 ^b^	-	-
9	Eucalyptol	12.66	1030	-	-	-	0.11 ± 0.01	-
10	*β*-Ocimene	13.57	1048	0.17 ± 0.01	-	-	-	-
11	*γ*-Terpinene	13.97	1057	-	0.12 ± 0.01	0.54 ± 0.01	0.05 ± 0.01	-
12	Terpinolene	15.31	1088	-	-	0.26 ± 0.02	-	-
13	(E)-Linalool oxide (furanoid)	15.35	1088	0.25 ± 0.02	-	-	-	-
14	Linalool	16.08	1100	68.64 ± 1.15 ^a^	-	-	-	-
15	Hotrienol	16.18	1107	0.62 ± 0.02	-	-	-	-
16	Cosmene	17.29	1132	0.12 ± 0.01	-	-	-	-
17	(+)-2-Bornanone	17.82	1140	-	-	2.02 ± 0.13 ^f^	-	-
18	Citronellal	18.42	1153	-	0.18 ± 0.03	-	-	-
19	Borneol	18.83	1162	-	-	0.46 ± 0.01	-	-
20	*p*-Mentha-1,5-dien-8-ol	18.94	1161	0.16 ± 0.01	-	-	-	-
21	Isoneral	18.95	1165	-	1.00 ± 0.09 ^g^	-	-	-
22	Terpinen-4-ol	19.39	1172	-	-	0.42 ± 0.06	-	-
23	3,6-Octadienal, 3,7-dimethyl-	19.80	1183	-	1.77 ± 0.12 ^ef^	-	-	-
24	*α*-Terpineol	20.02	1188	-	-	2.77 ± 0.33 ^e^	-	-
25	Nerol	21.83	1229	-	2.22 ± 0.11 ^e^	-	-	0.09 ± 0.01
26	Neral	22.44	1240	-	30.75 ± 0.09 ^b^	1.88 ± 0.17 ^f^	-	0.21 ± 0.02
27	Piperitone	22.89	1251	-	-	0.82 ± 0.03	-	-
28	Geraniol	23.17	1255	-	2.90 ± 0.11 ^d^	54.46 ± 0.56 ^a^	-	46.15 ± 0.12 ^a^
29	(E)-Citral	23.81	1270	-	35.80 ± 0.17 ^a^	0.52 ± 0.09	-	0.50 ± 0.06
30	Trans-Geranic acid methyl ester	26.13	1321	-	-	-	-	0.28 ± 0.03
31	Eugenol	27.48	1353	-	-	-	0.18 ± 0.02	-
32	*α*-Copaene	28.22	1372	-	0.47 ± 0.02	0.23 ± 0.01	0.07 ± 0.01	0.23 ± 0.02
33	(E)-Methyl cinnamate	28.61	1380	-	-	-	-	46.20 ± 0.44 ^a^
34	*β*-Cubebene	28.87	1387	-	-	-	-	0.25 ± 0.02
35	(-)-*β*-Elemene	28.96	1389	-	0.79 ± 0.08	-	-	0.07 ± 0.01
36	Methyleugenol	29.58	1408	-	-	-	1.50 ± 0.27 ^c^	0.05 ± 0.01
37	Caryophyllene	30.01	1415	1.69 ± 0.21 ^c^	1.49 ± 0.25 ^fg^	2.27 ± 0.26 ^f^	2.11 ± 0.19 ^b^	0.60 ± 0.07
38	Humulene	31.41	1445	0.10 ± 0.01	-	-	-	0.19 ± 0.03
39	Germacrene D	32.56	1478	-	-	-	0.09 ± 0.01	0.14 ± 0.01
40	*β*-Selinene	32.74	1482	-	-	2.84 ± 0.19 ^e^	-	-
41	*α*-Selinene	33.11	1493	-	-	0.78 ± 0.10	-	-
42	Bicyclogermacrene	33.18	1493	-	3.03 ± 0.25 ^d^	-	0.25 ± 0.03	-
43	*α*-Farnesene	33.80	1508	-	0.44 ± 0.02	-	-	-
44	*δ*-Cadinene	34.29	1521	-	0.28 ± 0.01	-	0.09 ± 0.01	0.48 ± 0.03
45	Elemol	35.32	1550	20.72 ± 0.73 ^b^	-	-	-	-
46	Elemicin	35.86	1558	-	-	-	94.56 ± 0.98 ^a^	-
47	Spatulenol	36.33	1571	-	10.62 ± 0.77 ^c^	-	-	-
48	Caryophyllene oxide	36.52	1576	-	3.21 ± 0.21 ^d^	0.72 ± 0.07	-	0.18 ± 0.01
49	*γ*-Eudesmol	38.32	1630	1.07 ± 0.11 ^d^	-	-	-	-
50	Isospathulenol	38.53	1640	-	0.88 ± 0.04	-	-	-
51	*β*-Selinenol	38.86	1649	1.45 ± 0.28 ^cd^	-	-	-	-
52	Farnesol	40.63	1721	-	-	-	0.26 ± 0.05	1.64 ± 0.09 ^b^
53	Hexanedioic acid, bis(2-ethylhexyl) ester	48.51	2398	-	0.23 ± 0.01	0.56 ± 0.01	-	-
	Total			94.73 ± 0.66	97.48 ± 0.87	97.8 ± 1.13	99.27 ± 0.15	97.31 ± 0.56
	NO of compounds			12	22	22	15	17
	EO yields (%)			0.68	0.49	0.77	0.39	1.02

Note: RT, retention time; RI, retention indices; Values are expressed as the average of three parallel experiments ± standard deviation; ‘-’ indicates not detected. Compound identification was based on the NIST mass spectrometry database and RI values, with several compounds identified using reliable standardized compounds. Means in a row with different letters indicate statistical significance (*p* < 0.05). Components with Area (%) ≥ 5% are considered major components.

**Table 2:** Total polyphenol content and antioxidant capacity of *C. tenuipilis* essential oils.

Numbers	ABTS (µmol TE/g DW)	DPPH (µmol TE/g DW)	FRAP (µmol TE/g DW)	TAC (% Inhibition)	TP (mg GAE/g DW)
BX07	20.82 ± 1.75 ^e^	25.65 ± 0.57 ^e^	1.33 ± 0.67 ^e^	28.90 ± 3.22 ^e^	35.85 ± 1.13 ^e^
FH01	52.09 ± 0.66 ^c^	75.99 ± 1.65 ^b^	4.53 ± 0.54 ^b^	83.83 ± 3.92 ^b^	153.8 ± 1.58 ^b^
FH07	62.33 ± 0.49 ^a^	49.55 ± 1.01 ^c^	3.71 ± 0.16 ^d^	72.73 ± 13.94 ^c^	113.4 ± 1.87 ^c^
YC02	57.27 ± 0.82 ^b^	83.75 ± 0.15 ^a^	5.87 ± 0.13 ^a^	97.29 ± 2.71 ^a^	239.4 ± 6.87 ^a^
YC04	45.33 ± 0.60 ^d^	40.44 ± 0.85 ^d^	3.79 ± 0.42 ^c^	61.86 ± 7.18 ^d^	80.01 ± 1.07 ^d^
*p*-value	*p* < 0.01	*p* < 0.01	*p* < 0.01	*p* < 0.05	*p* < 0.01

Note: ABTS refers to 2,20-Azinobis-(3-ethylbenzthiazoline-6-sulphonate); DPPH denotes 2,2-diphenyl-1-picrylhydrazyl radical scavenging activity; FRAP stands for ferric reducing antioxidant power; TAC indicates total antioxidant capacity; TP indicates total polyphenol. Values are presented as mean ± standard deviation. The letters a, b, c, d, e in the same row indicates statistically significant differences determined by one-way ANOVA and LSD test, with *p* < 0.05.

**Table 3:** MIC and MBC of *C. tenuipilis* essential oils against bacteria.

Bacteria		*C. tenuipilis* Essential Oils (μL/mL)					Antibiotic (μg/mL)
		BX07	FH01	FH07	YC02	YC04	Ciprofloxacin
*B. cereus*	MIC	6.25	3.13	6.25	12.5	6.25	0.25
	MBC	12.5	6.25	12.5	25	12.5	0.25
*B. subtilis*	MIC	6.25	3.13	12.5	12.5	6.25	0.13
	MBC	12.5	6.25	12.5	25	>25	0.25
*E. coli*	MIC	12.5	6.25	12.5	12.5	12.5	0.06
	MBC	12.5	6.25	>25	25	12.5	0.13
*S. aureus*	MIC	3.13	6.25	12.5	>25	12.5	0.50
	MBC	6.25	12.5	12.5	>25	12.5	1

Note: MIC, minimum inhibitory concentration; MBC, minimum bactericidal concentration, which is the lowest concentration that kills 99.9% of the bacterial population. The results reflect the potency of *C. tenuipilis* EOs and ciprofloxacin against four bacteria, offering important information for further pharmacological and therapeutic applications.

## Data Availability

The original contributions presented in this study are included in the article. Further inquiries can be directed at the corresponding author.

## Supplementary Materials

The following supporting information can be downloaded at: https://www.mdpi.com/article/10.3390/plants14223409/s1, Figure S1. GC-MS of five EOs; Figure S2. Curve fitting.

## Abbreviations

The following abbreviations are used in this manuscript:

ABTS	2,2′-Azino-bis-3-ethylbenzthioazoline-6-sulfonic acid
ANOVA	Analysis of Variance
BHA	Butylated hydroxyanisole
BHT	Butylated hydroxytoluene
CCK-8	Cell Counting Kit-8
CFU	Colony Forming Unit
DPPH	2,2-Diphenyl-1-picrylhydrazyl
DIZ	Diameter of the Inhibitory Zone
DMEM	Dulbecco’s Modified Eagle Medium
DMSO	Dimethyl Sulfoxide
EO	Essential Oil
FRAP	Ferric-Reducing Antioxidant Power
GAE	Gallic Acid Equivalents
GC-MS	Gas Chromatography–Mass Spectrometry
IC50	Half Maximal Inhibitory Concentration
INT	p-Iodonitrotetrazolium violet
LSD	Least Significant Difference
MBC	Minimum Bactericidal Concentration
MIC	Minimum Inhibitory Concentration
NIST	National Institute of Standards and Technology
RI	Retention Indices
RT	Retention Time
TAC	Total Antioxidant Capacity
TE	Trolox Equivalents
TP	Total Polyphenol
