# Supplementary figures and images for: Study on Chemical Diversity, Antioxidant and Antibacterial Activities, and HaCaT Cytotoxicity of Camphora tenuipilis (a Traditional Aromatic Plant from Xishuangbanna)
Source: Plants (Basel). 2025 Nov 7;14(22):3409. doi: 10.3390/plants14223409 (PMC12656405; doi:10.3390/plants14223409)

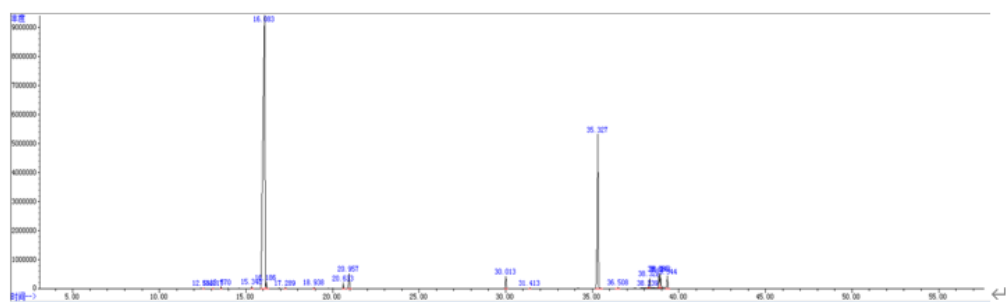

BX07

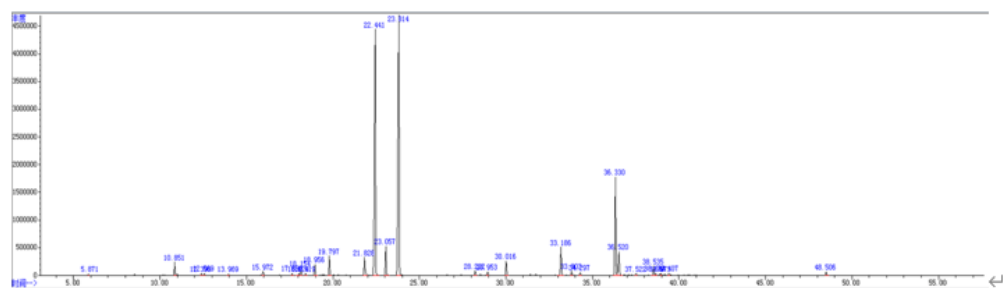

FH01

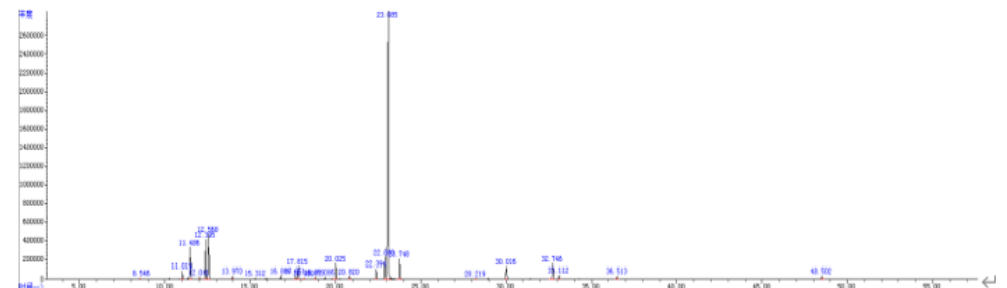

FH07

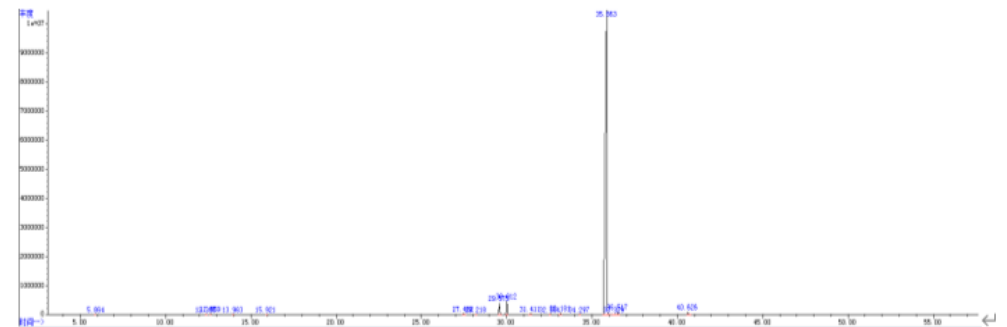

YC02

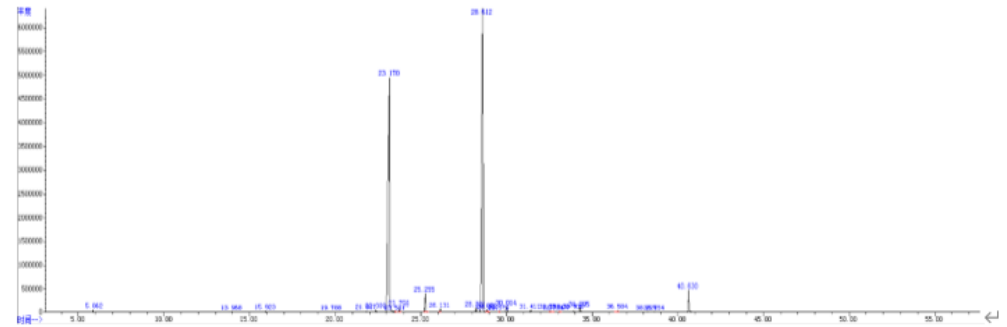

YC04

Figure S1. GC-MS of five EOs

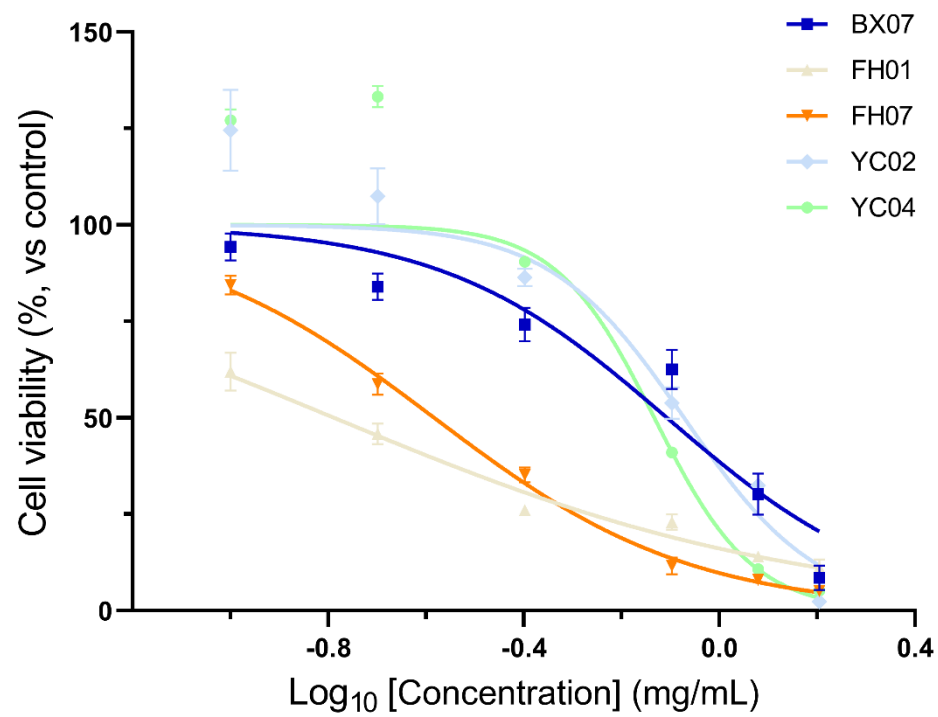

Figure S2. Curve fitting

Supplement: Supplementary file 1 [file plants-14-03409-s001.zip › plants-3923242-supplementary.pdf]
